# Targeting the multifaceted BRAF in cancer: New directions

**DOI:** 10.18632/oncotarget.28612

**Published:** 2024-07-16

**Authors:** Eamon Toye, Alexander Chehrazi-Raffle, Justin Hwang, Emmanuel S. Antonarakis

**Affiliations:** ^1^Masonic Cancer Center, University of Minnesota-Twin Cities, Minneapolis, MN 55455, USA; ^2^Department of Medicine, University of Minnesota-Twin Cities, Minneapolis, MN 55455, USA; ^3^Perelman School of Medicine, University of Pennsylvania, Philadelphia, PA 19146, USA; ^4^City of Hope Comprehensive Cancer Center, Duarte, CA 91010, USA

**Keywords:** *BRAF*, MAPK, pan-cancer, precision oncology, genomics

## Abstract

Activating mutations in the mitogen-activated protein kinase (MAPK) pathway represent driver alterations governing tumorigenesis, metastasis, and therapy resistance. MAPK activation predominantly occurs through genomic alterations in *RAS* and *BRAF*. BRAF is an effector kinase that functions downstream of *RAS* and propagates this oncogenic activity through MEK and ERK. Across cancers, *BRAF* alterations include gain-of-function mutations, copy-number alterations, and structural rearrangements. In cancer patients, BRAF-targeting precision therapeutics are effective against Class I *BRAF* alterations (p.V600 hotspot mutations) in tumors such as melanomas, thyroid cancers, and colorectal cancers. However, numerous non-Class I BRAF inhibitors are also in development and have been explored in some cancers. Here we discuss the diverse forms of *BRAF* alterations found in human cancers and the strategies to inhibit them in patients harboring cancers of distinct origins.

## INTRODUCTION

### BRAF is an effector of MAPK signaling

The mitogen-activated protein kinase (MAPK) pathway is a vital cell signaling pathway [1]. The MAPK pathway regulates cellular functions ranging from cell growth and proliferation to tissue repair and wound healing [2–4]. Functional members of this pathway are the cytoplasmic serine/threonine kinases *RAS*, RAF, MEK, and ERK. Canonical activation of receptor tyrosine kinases leads to *RAS*-GDP phosphorylation, creating activated *RAS*-GTP. This in turn phosphorylates RAF, which dimerizes and then phosphorylates MEK1/2. Phosphorylated MEK1/2 then activates ERK1/2, which regulates cellular growth, proliferation, and cell death [5–7]. There are three distinct isoforms of the RAF proteins encoded by the *ARAF, BRAF,* and *RAF1 (*also known as *CRAF*) genes [8]. Each of these isoforms contains the same conserved regions (CR): the *RAS*-binding domain (CR1), regulatory domain (CR2), and functional kinase domain (CR3) [9]. Although all RAF kinases promote MAPK signaling, *BRAF* is the most consistently altered RAF family gene across human cancers.

### BRAF in cancer

**Figure 1:**
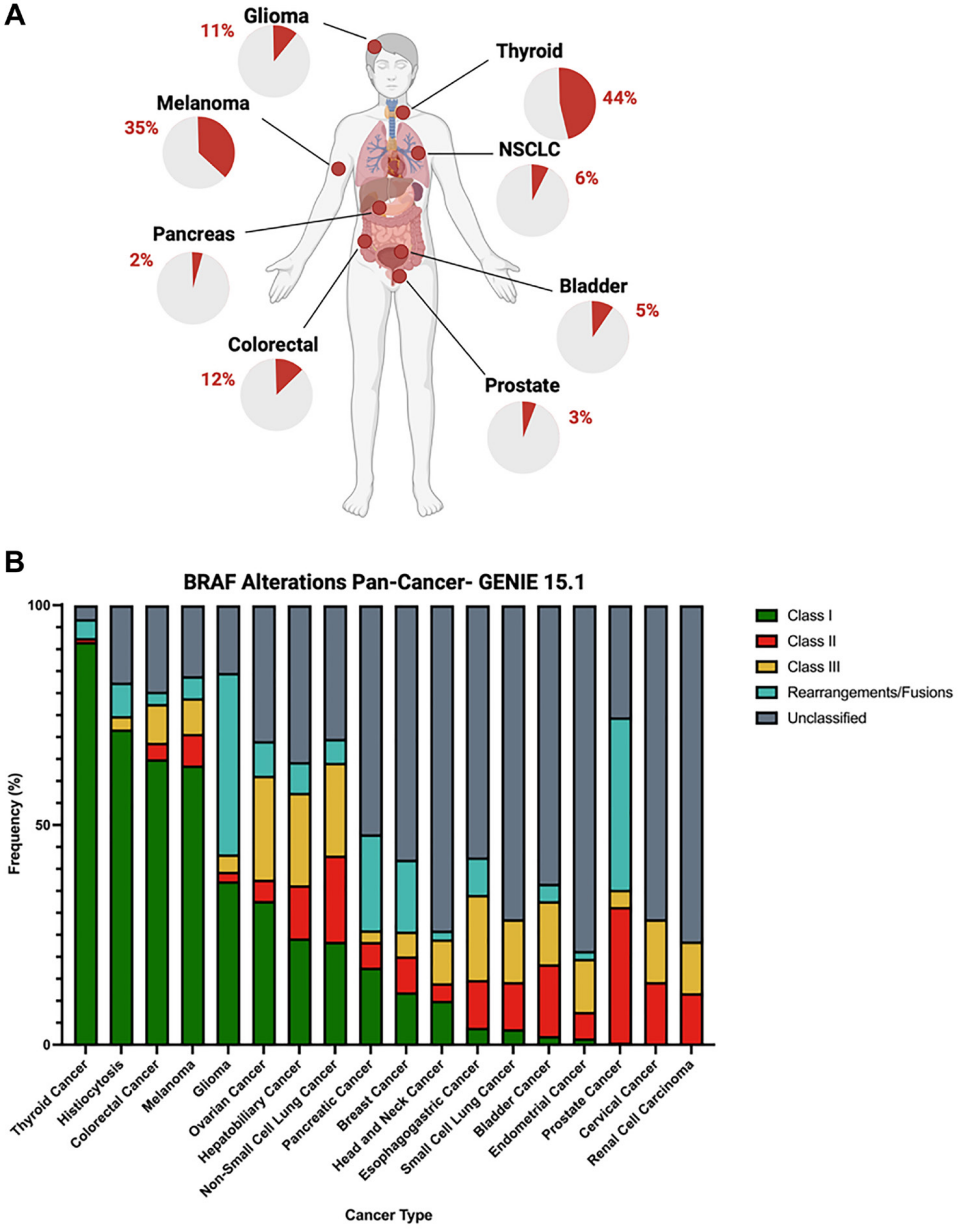
(**A**) *BRAF* alteration frequencies, according to GENIE 15.1, among select solid organ tumors (glioma, 11%; thyroid cancer, 44%; non-small cell lung cancer, 6%; bladder cancer, 5%; prostate cancer, 3%; colorectal cancer, 12%; pancreatic cancer, 2%; and melanoma, 35%). (**B**) Relative proportions of distinct classifications of *BRAF* alterations across multiple cancer types (green = Class I, red = Class II, yellow = Class III, teal = structural rearrangement, and gray = unclassified).

Due to these pro-growth and pro-survival functions, cancer cells can develop “oncogenic addiction” towards BRAF’s signaling activity [10, 11]. Initially characterized in melanomas, various groups including ours have identified activating *BRAF* alterations across multiple cancers [12–19]. Based on the American Association for Cancer Research’s (AACR) Genomics Evidence Neoplasia Information Exchange (GENIE, version 15.1) dataset, *BRAF* alterations are observed in several human malignancies, including 44% of thyroid cancers, 35% of melanomas, and 12% of colorectal cancers; with multiple other major cancer types exhibiting lower frequencies of *BRAF* alterations (Figure 1A). Consistent with these genomic observations, abundant pre-clinical evidence across many malignancies indicates that *BRAF* alterations drive an increase in MAPK signaling and, thus, tumor growth in cancer cell lines and transgenic mouse models [20–22].

### Classes of *BRAF* alterations in cancer

Studies generally recognize three classes of activating *BRAF* alterations defined by their mechanisms of *RAS* dependency, dimerization status, and kinase activity [23]. Class I *BRAF* mutants involve missense mutations at valine 600 (p.V600), which lead to *RAS*-independent BRAF monomeric activation with strongly elevated kinase activity [12, 24]. Class I alterations are the dominant form of *BRAF* alteration in melanomas, thyroid, colorectal, and ovarian cancers (Figure 1B). In these cancer types, tumors with Class I alterations can be treated with BRAF inhibitors or combinations of BRAF/MEK inhibitors. Class II *BRAF* alterations consist of non-p.V600 mutations and structural rearrangements that yield homodimers, that are also *RAS*-independent but have moderate-to-high kinase activity [25]. Class II alterations appear to be the predominant form of *BRAF* alteration in prostate, bladder, and non-small cell lung cancers. Class III *BRAF* alterations are also non-p.V600 mutants, however, they heterodimerize with ARAF or wild-type BRAF but are *RAS*-dependent and have low kinase activity [26]. Cervical cancer, hepatobiliary cancer, and non-small cell lung cancer harbor relatively high rates of Class III *BRAF* alterations. Due to the requisite upstream stimulus of mutant *RAS*, Class III mutants may be susceptible to *RAS*-specific targeted therapies [27].

### Evolution of BRAF inhibition in the clinic

**Figure 2:**
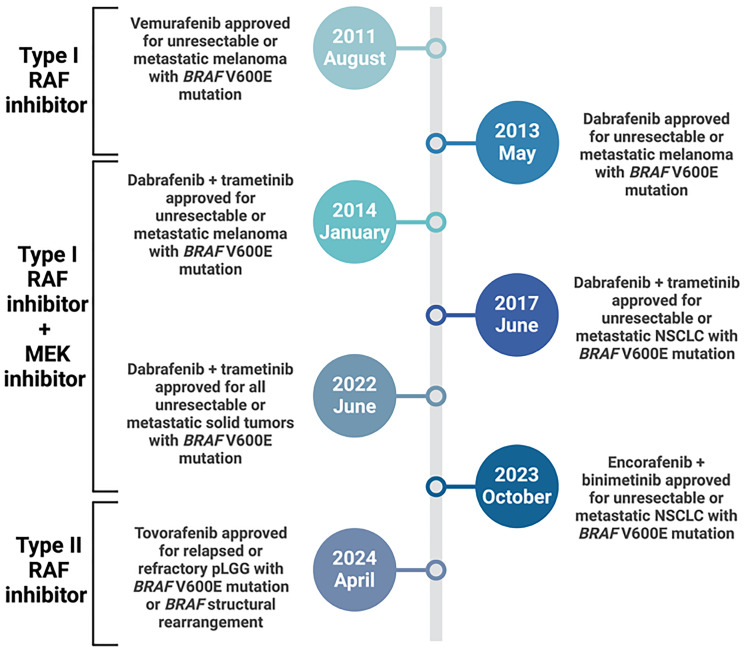
Timeline showing major FDA approvals involving BRAF/MEK inhibitors. pLGG = pediatric low-grade gliomas NSCLC = non small cell lung cancer.

Over the past 15 years, precision therapies targeting *BRAF* have been used to treat patients with metastatic melanomas. In 2010, Flaherty et al. showed an objective response in >80% of Class I-altered metastatic melanoma patients using PLX4032, later named vemurafenib [28]. In 2012, Hauschild et al. demonstrated a 70% reduction in the risk of death (HR = 0.30) in *BRAF* p.V600E-mutated metastatic melanoma patients treated with dabrafenib, compared to chemotherapy (dacarbazine) control [29]. These studies were foundational for establishing the utility of the Class I BRAF inhibitors, vemurafenib and dabrafenib. Flaherty et al. also indicated that metastatic melanomas resistant to BRAF inhibitors still require MAPK activity and that combined use of BRAF and MEK inhibitors demonstrated a significant increase in median progression-free survival (PFS) compared to BRAF monotherapy [30, 31]. In 2022, the FDA granted accelerated approval for this combination in all Class I *BRAF*-mutant metastatic solid tumors [32]. In sum, inhibitors targeting Class I *BRAF* alterations represent one of the major successes in precision oncology to date (Figure 2).

In contrast, agents targeting Class II and III *BRAF* alterations have yet to achieve similar success. Mechanistically, Class II and III *BRAF* alterations are not susceptible to Class I inhibitors, which attenuate BRAF activity by binding monomeric BRAF and inducing dimerization. Paradoxically, these drugs enhance Class II and III altered BRAF signaling, in which dimerization promotes enhanced MAPK signaling and ultimately tumor growth [25, 33–35]. Recently, a new focus has been on developing paradox breakers and type II pan-RAF inhibitors that ablate the upregulated MAPK activity seen in Class II and III *BRAF*-altered cancers treated with currently FDA-approved RAF inhibitors. Results from the phase II FIREFLY-1 clinical trial assessing the type-II pan-RAF inhibitor tovorafenib showed high tolerability and strong efficacy in non-Class I *BRAF-*mutant pediatric low-grade gliomas (pLGG) harboring *BRAF* structural rearrangements [36]. Specifically, relapsed/refractory pLGG patients demonstrated an overall response rate of 67% and a median duration of response of 16.6 months, exceeding both the primary and secondary pre-specified endpoints. With these striking results, the FDA recently approved tovorafenib for relapsed/refractory pLGG patients harboring *BRAF* structural rearrangements [37].

### *BRAF* structural rearrangements

Structural rearrangements of *BRAF* also represent a potentially actionable subgroup of *BRAF* alterations and are most prevalent in gliomas, prostate cancers and pancreatic cancers (Figure 1B). In one case study, a prostate cancer patient with an *SND1-BRAF* structural rearrangement who was refractory to standard therapies showed a strong clinical response to trametinib monotherapy, an FDA-approved MEK inhibitor [38]. These *BRAF* rearrangement events were also seen with some frequency at the prostate cancer level, as our group recently examined 15,864 prostate tissue biopsies and 7,566 liquid biopsies, where we showed that 46.7% of prostate cancer patients harboring *BRAF* alterations exhibited structural rearrangements [13].

**Figure 3:**
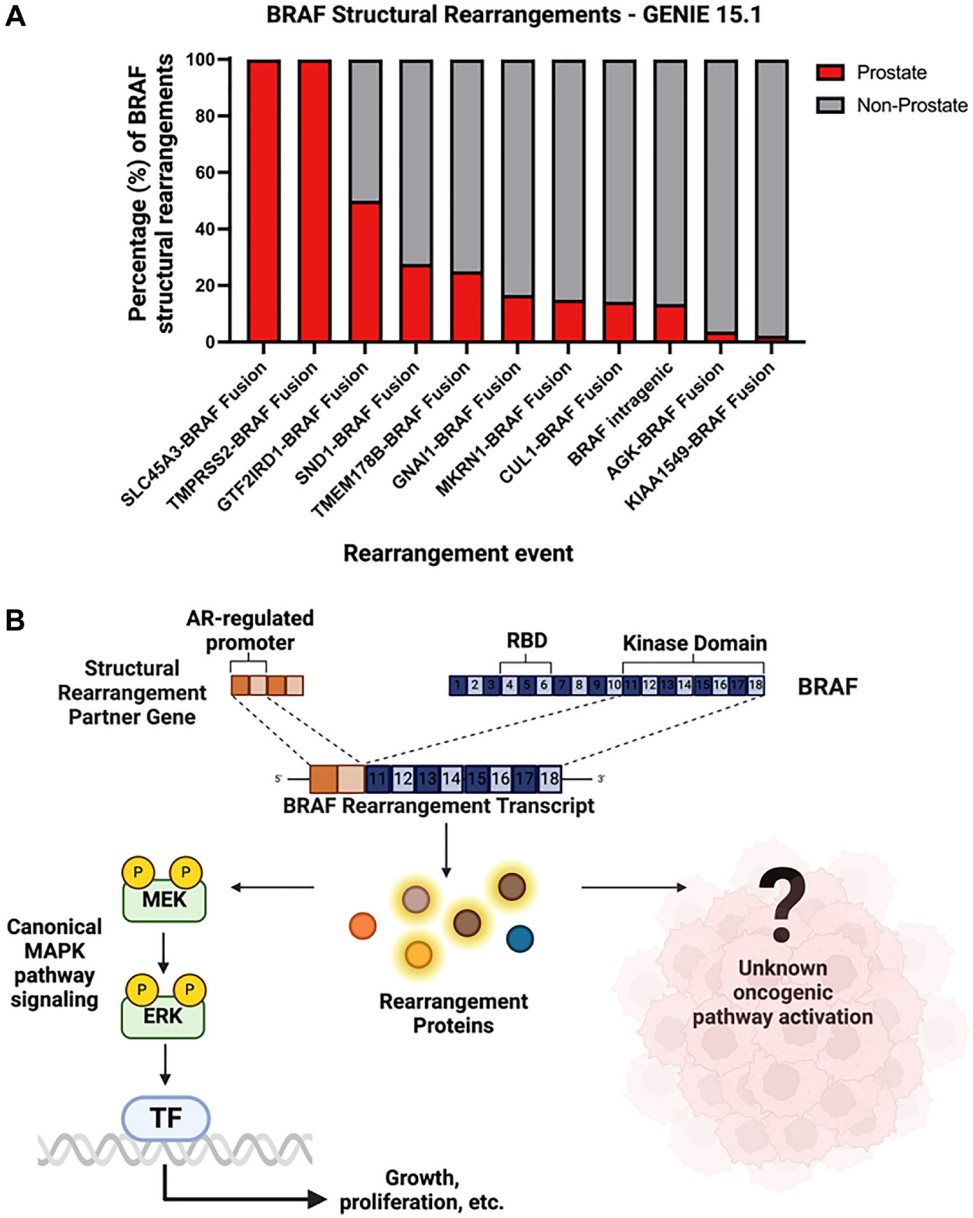
(**A**) Relative proportion of specific structural rearrangement *BRAF* events in prostate cancer versus all other cancer types (red = prostate cancer, gray = non-prostate cancer). (**B**) Mechanism depicting possible downstream effects of *BRAF* structural rearrangement events that yield a truncated *BRAF* transcript with a fully functional kinase domain.

In addition, certain structural rearrangements were exclusively found in specific cancer types, as is the case for *SLC45A3-BRAF* and *TMPRSS2-BRAF* structural rearrangements in prostate tumors and no other cancer types (Figure 3A). *TMPRSS2* and *SLC45A3* are generally expressed in the prostate epithelium and their expression is regulated by the lineage-specifying transcriptional activity of the androgen receptor (AR) [39]. In prostate cancers, *SLC45A3-BRAF* and *TMPRSS2-BRAF* structural rearrangements thus result in activated BRAF kinase activity under the control of AR (Figure 3B). This fusion-protein product would also be predicted to be independent of *RAS* activity due to loss of the 5′ *RAS*-binding domain. Altogether, this represents another mechanism of BRAF activation in cancer, whereby BRAF kinase expression is driven by aberrant transcriptional activity. The efficacy of BRAF or MAPK inhibitors in such cancer patients requires further investigation in prospective studies.

## CONCLUSION

Class I BRAF inhibitors are one of the landmark achievements in precision oncology, as recently evidenced by the tissue-agnostic FDA approval of dabrafenib/trametinib in patients with metastatic *BRAF* p.V600E-mutant solid tumors. Although targeted therapies against Class II alterations, Class III mutations, and *BRAF* rearrangements are largely still in early development, the accelerated approval of tovorafenib for patients with relapsed/refractory *BRAF*-altered pediatric low-grade glioma underscores the therapeutic potential of this and other next-generation strategies to target aberrant MAPK signaling.
